# KIT及其他克隆性基因突变对核心结合因子相关急性髓系白血病的预后价值

**DOI:** 10.3760/cma.j.issn.0253-2727.2021.08.006

**Published:** 2021-08

**Authors:** 天梅 吴, 胜利 薛, 正 李, 景秋 于, 隽 王, 斌儒 王, 超玲 万, 向东 申, 桥成 邱, 协炳 鲍, 德沛 吴

**Affiliations:** 苏州大学附属第一医院、江苏省血液研究所、国家血液系统疾病临床医学研究中心、国家卫生健康委员会血栓与止血重点实验室，苏州 215006 The First Affiliated Hospital of Soochow University, Jiangsu Institute of Hematology, National Clinical Research Center for Hematologic Diseases, NHC Key Laboratory of Thrombosis and Hemostasis, Suzhou 215006, China

**Keywords:** 核心结合因子, 白血病，髓样，急性, 基因突变, 基因，KIT, Core binding factors, Leukemia, myeloid, acute, Genetic mutations, Gene, KIT

## Abstract

**目的:**

评价基于二代测序（NGS）检测技术下的克隆性基因突变对核心结合因子相关急性髓系白血病（CBF-AML）预后的影响。

**方法:**

回顾性分析2011年7月至2017年8月在苏州大学附属第一医院血液科诊治的195例成人初治CBF-AML患者，其中诱导化疗达完全缓解的患者190例，包括134例RUNX1-RUNXIT1^+^ AML和56例CBFβ-MYH11^+^ AML，年龄15～64岁，中位随访时间43.6个月。采用Log-rank检验和Cox回归模型分析临床因素和基因突变对患者总生存（OS）和无病生存（DFS）的影响。

**结果:**

在195例患者中，KIT基因突变发生率最高（47.6％），其次为NRAS（20.0％）、FLT3（18.4％）、ASXL2（14.3％）、KRAS（10.7％）、ASXL1（9.7％）。按基因功能分类，酪氨酸激酶信号通路基因突变发生率最高（76.4％），其次为染色质修饰相关基因（29.7％）。在接受强化巩固治疗的患者中，CBFβ-MYH11^+^ AML患者的OS有优于RUNX1-RUNXIT1^+^ AML患者的趋势（*P*＝0.062）。染色质修饰相关基因突变仅在RUNX1-RUNXIT1^+^ AML中检出，但对患者的DFS无明显影响（*P*＝0.557）。染色质修饰相关基因突变阳性且接受异基因造血干细胞移植（allo-HSCT）的患者预后最好。多因素分析显示KIT exon17突变为影响RUNX1-RUNXIT1^+^ AML患者DFS的独立危险因素（*P*<0.001），allo-HSCT能明显改善RUNX1-RUNXIT1^+^ AML患者的DFS（*P*＝0.010）。

**结论:**

合并KIT exon17突变的RUNX1-RUNXIT1^+^ AML患者预后差，allo-HSCT可改善这部分患者的预后，allo-HSCT也能使染色质修饰相关基因突变阳性患者的预后得到改善。

## Abstract (en)

**Objective:**

To evaluate the prognostic significance of clonal gene mutations using next-generation sequencing in patients with core-binding factor acute myeloid leukemia（CBF-AML）who achieved first complete remission after induction chemotherapy.

**Methods:**

The study, which was conducted from July 2011 to August 2017 in First Affiliated Hospital of Soochow University, comprised 195 newly diagnosed patients with CBF-AML, including 190 patients who achieved first complete remission after induction chemotherapy. The cohort included 134 patients with RUNX1-RUNXIT1^+^ AML and 56 patients with CBFβ-MYH11^+^ AML. The cohort age ranged from 15 to 64 years, with a median follow-up of 43.6 months. Overall survival（OS）and disease-free survival（DFS）were assessed by the log-rank test, and the Cox proportional hazards regression model was used to determine the effects of clinical factors and genetic mutations on prognosis.

**Results:**

The most common genetic mutations were in KIT （47.6％）, followed by NRAS（20.0％）, FLT3（18.4％）, ASXL2（14.3％）, KRAS（10.7％）, and ASXL1（9.7％）. The most common mutations involved genes affecting tyrosine kinase signaling（76.4％）, followed by chromatin modifiers（29.7％）. Among the patients receiving intensive consolidation therapy, the OS tended to be better in patients with CBFβ-MYH11^+^ AML than in those with RUNX1-RUNXIT1^+^ AML（*P*＝0.062）. Gene mutations related to chromatin modification, which were detected only in patients with RUNX1-RUNXIT1^+^ AML, did not affect DFS（*P*＝0.557）. The patients with mutations in genes regulating chromatin conformation who received allo-hematopoietic stem cell transplantation（allo-HSCT）achieved the best prognosis. Multivariate analysis identified KIT exon 17 mutations as an independent predictor of inferior DFS in patients with RUNX1-RUNXIT1^+^ AML（*P*<0.001）, and allo-HSCT significantly prolonged DFS in these patients（*P*＝0.010）.

**Conclusion:**

KIT exon 17 mutations might indicate poor prognosis in patients with RUNX1-RUNXIT1^+^ AML. Allo-HSCT may improve prognosis in these patients, whereas allo-HSCT might also improve prognosis in patients with mutations in genes related to chromatin modifications.

急性髓性白血病（AML）是一组常见的具有高度异质性的恶性克隆性血液病。染色体t（8;21）（q21;q22）和inv（16）（p13;22）/t（16;16）（p13;q22）是AML中常见的重现性细胞遗传学异常，分别形成RUNX1-RUNXIT1和CBFβ-MYH11融合基因。由RUNX1和CBFβ组成的核心结合因子（CBF）蛋白复合物对于髓系分化至关重要，上述两种重排均会破坏CBF功能，导致髓样分化受阻，最终导致CBF-AML。CBF-AML占成人初治AML的10％～15％，患者有较好的临床预后[1]–[3]。然而45％的CBF-AML患者在标准化疗后复发[4]–[6]，且多个研究表明CBF-AML患者间存在异质性[7]–[11]。超过80％的CBF-AML患者可出现KIT、FLT3、NRAS及KRAS基因突变，这些基因突变可能通过促进增殖充当致白血病的协作因子，通常与不良的临床预后相关[12]–[13]。近年来，随着二代测序技术（NGS）的广泛应用，基于NGS的分子遗传学检测在AML患者诊治中的临床价值已被逐渐认识。本研究收集了我院收治的195例成人初治CBF-AML患者的临床资料，评价KIT等基因突变对CBF-AML临床预后的影响，以进一步优化CBF-AML的治疗选择。

## 病例与方法

一、病例

纳入2011年7月至2017年8月在苏州大学附属第一医院血液科诊治的195例初治的CBF-AML患者（年龄15～64岁）。患者的入选标准：RUNX1-RUNXIT1^+^ AML或CBFβ-MYH11^+^ AML诊断符合WHO标准[14]；既往无血液系统疾病或肿瘤疾病史；美国东部肿瘤协作组（ECOG）体能评分<2分；无严重器官功能不全合并症；染色体核型分析和NGS资料完整。其中诱导化疗达完全缓解（CR）状态的190例患者用于预后分析，包括134例RUNX1-RUNXIT1^+^ AML和56例CBFβ-MYH11^+^ AML患者。这些患者接受1～2个疗程标准诱导化疗方案达第1次CR（CR_1_），并接受高剂量化疗或自体造血干细胞移植（auto-HSCT）（两者统称为强化巩固治疗）或异基因造血干细胞移植（allo-HSCT）。10例患者在强化巩固治疗期间出现复发并接受allo-HSCT治疗，本研究将这部分患者纳入接受强化巩固治疗组，随访时间截至allo-HSCT前，结局按照删失处理。

二、研究方法

1. MICM分型：完善骨髓细胞形态学、流式细胞术免疫分型、染色体核型、43种融合基因筛查。

2. NGS：利用Ion Torrent S5 NGS测序平台（美国Thermo Fisher公司产品）采用靶向扩增子法对获得足够数量基因组DNA的样本进行49个白血病相关基因靶向高通量基因测序。NGS测序结果由Ion Torrent S5仪器自带软件和插件进行初步分析，得到该540芯片上样率、人类基因组参考序列匹配度、整体数据量和中位测序长度等，在靶率97％～99％，平均深度2 000×，均一度94％～97％，中位测序长度195 bp。并用PCR-毛细管电泳法检测FLT3-ITD突变，Sanger测序法检测CEBPA CDS区和NPM1第12号外显子突变。

3. 治疗方案：所有患者依照成人AML中国诊疗指南（2017年版）[15]，接受阿糖胞苷联合去甲氧柔红霉素（“7+3”方案：阿糖胞苷100 mg/m^2^第1～7天，去甲氧柔红霉素8或10或12 mg/m^2^第1～3天）为基础的标准诱导化疗方案，诱导化疗达到CR_1_后采用大剂量阿糖胞苷（3 g/m^2^，每12 h 1次，6个剂量）3～4个疗程或结合患者意愿及经济状况选择性进行auto-HSCT或allo-HSCT治疗。

4. 随访及指标定义：采用电话、门诊随诊、医院病例登记系统等方式进行随访。随访时间截至2020年9月1日，总生存（OS）时间定义为从疾病确诊至患者死亡或末次随访日期。无病生存（DFS）时间定义为从患者化疗后达到CR_1_至疾病复发、患者死亡或末次随访日期。复发定义为在达到CR后骨髓中再次出现>5％原始细胞，或外周血中出现任何比例的原始细胞，或髓外复发。基因突变阳性定义为突变比例≥1％。

三、统计学处理

采用IBM SPSS 22.0软件进行统计分析，计量资料符合正态分布用*x*±*s*描述；不符合正态分布用*M*（范围）描述，计数资料以百分率表示。方差齐时使用*t*检验，否则使用非参数秩和检验。分类资料采用*χ*^2^检验或Fisher精确检验。生存曲线采用Kaplan-Meier法描绘，单因素生存分析使用Log-rank检验进行组间比较。将单因素分析中*P*<0.1的变量纳入Cox回归模型进行多因素分析。所有统计检验以*α*＝0.05，*P*<0.05为差异有统计学意义。

## 结果

一、一般临床特征

纳入成人初治CBF-AML患者共195例，中位年龄39（15～64）岁，其中男121例，女74例。CBF-AML患者初诊时的一般临床特征、MICM分型和发生频率≥3％的相对常见基因突变特征见表1。

**表1:** 195例核心结合因子相关急性髓系白血病（CBF-AML）患者一般临床特征和基因突变特征

项目	CBF-AML（195例）	RUNX1-RUNX1T1^+^ AML（139例）	CBFβ-MYH11^+^ AML（56例）	*P*值
性别（例，男/女）	121/74	85/54	36/20	0.683
年龄［岁，*M*（范围）］	39（15～64）	38（15～62）	39（16～64）	0.438
WBC［×10^9^/L，*M*（范围）］	11.3（0.8～178.2）	9.0（0.8～79.1）	30.1（2.4～178.2）	<0.001
HGB［g/L，*M*（范围）］	74.0（32.0～155.0）	72.0（32.0～155.0）	76.5（44.0～150.0）	0.109
PLT［×10^9^/L，*M*（范围）］	28（4～201）	29（4～201）	27（7～198）	0.470
LDH［U/L，*M*（范围）］	412（72～3036）	443（72～3036）	406（107～1411）	0.060
外周血原始细胞［％，*M*（范围）］	39.5（2～90）	38.5（2～87）	42（7～90）	0.298
骨髓原始细胞［％，*M*（范围）］	46.0（5.5～98.0）	43.0（5.5～98.0）	53.5（13.5～87.0）	0.107
CD19阳性［例数/总例数（％）］	93/191（48.7）	93/135（68.9）	0/56（0）	<0.001
染色体核型［例（％）］				
单独染色体易位	88（45.1）	51（36.6）	37（66.1）	<0.001
附加LOS	68（34.8）	68（48.9）	0（0）	<0.001
−Y	51（26.1）	51（36.6）	0（0）	<0.001
−X	17（8.7）	17（12.2）	0（0）	0.004
附加del（9q）	11（5.6）	11（7.9）	0（0）	0.036
附加+22	12（6.1）	1（0.7）	11（19.6）	<0.001
附加+8	6（3.0）	1（0.7）	5（8.9）	0.001
基因突变［例（％）］				
酪氨酸激酶信号通路				
KIT	93（47.6）	66（47.4）	27（48.2）	0.926
KITexon8	27（13.8）	12（8.6）	15（26.8）	0.001
KITexon17	75（38.4）	57（41.0）	18（32.1）	0.250
KITD816	43（22.0）	29（20.8）	14（25.0）	0.528
KITN822	35（17.9）	30（21.5）	5（8.9）	0.037
FLT3	36（18.4）	23（16.5）	13（23.2）	0.278
FLT3-ITD	11（5.6）	9（6.4）	2（3.6）	0.403
FLT3-TKD	21（10.7）	11（7.9）	10（17.9）	0.053
NRAS	39（20.0）	15（10.7）	24（42.9）	<0.001
KRAS	21（10.7）	7（5.0）	14（26.8）	<0.001
JAK1	6（3.0）	6（4.3）	0（0）	0.180
JAK2	4（2.0）	4（2.8）	0（0）	0.580
JAK3	10（5.1）	10（7.1）	0（0）	0.065
CSF3R	10（5.1）	9（6.4）	1（1.8）	0.325
CBL	7（3.5）	5（3.5）	2（3.6）	1.000
PTPN11	4（2.0）	0（0）	4（7.1）	0.006
染色质修饰				
ASXL1	19（9.7）	19（13.6）	0（0）	0.002
ASXL2	28（14.3）	28（20.1）	0（0）	<0.001
EZH2	12（6.1）	12（8.6）	0（0）	0.020
SETD2	5（2.5）	5（3.5）	0（0）	0.324
DNA甲基化				
TET2	11（5.6）	10（7.1）	1（1.8）	0.255
DNMT3A	3（1.5）	1（0.7）	2（3.6）	0.412
肿瘤抑制因子				
WT1	13（6.6）	6（4.3）	7（12.5）	0.079
转录因子				
RUNX1	5（2.5）	3（2.1）	2（3.6）	0.949

注：LOS：性染色体缺失

二、分子遗传学特征

本研究中，195例CBF-AML患者中有107例（54.8％）合并附加染色体异常，RUNX1-RUNXIT1^+^ AML发生率高于CBFβ-MYH11^+^ AML（63.3％对33.9％，*P*<0.001），两种CBF-AML亚型常见染色体异常分布具有明显的异质性。RUNX1-RUNXIT1^+^ AML最常见的附加染色体异常为性染色体缺失（LOS）和del（9q），CBFβ-MYH11^+^ AML最常见的附加染色体异常为+22和+8。

195例CBF-AML患者均接受二代基因测序，其中175例（90.8％）合并突变阳性。基因突变发生率由高到低分别涉及酪氨酸激酶信号通路（76.4％）、染色质修饰（29.7％）、转录因子（9.2％）、DNA甲基化（8.7％）和肿瘤抑制因子（8.2％）五个功能分类（表1）。最常见的基因突变是KIT突变（47.6％），其次为NRAS（20.0％）、FLT3（18.4％）、ASXL2（14.3％）、KRAS（10.7％）、ASXL1（9.7％）。不同CBF-AML亚型的突变状态有所差异。在RUNX1-RUNXIT1^+^ AML中，JAK、ASXL1、ASXL2突变更频繁。相比之下，CBFβ-MYH11 ^+^ AML中NRAS、KRAS和FLT3-TKD突变更常见。

三、KIT突变对CR率和预后的影响

190例达CR_1_状态的CBF-AML患者中接受去甲氧柔红霉素8、10、12 mg/m^2^诱导方案者分别为65、79和46例，后续行高剂量化疗88例，auto-HSCT 20例，allo-HSCT 82例。中位随访43.6（1.2～121.8）个月，随访过程中有30例（15.8％）患者复发，61例（32.1％）患者死亡。治疗方案和结局的分布情况在两种CBF-AML亚型中差异无统计学意义。总人群的3年OS率和DFS率分别为72.8％（95％*CI* 67.0％～79.8％）和65.6％（95％*CI* 59.3％～72.9％）。两种CBF-AML亚型患者的OS和DFS差异无统计学意义（OS，*P*＝0.302；DFS，*P*＝0.630），按治疗方案分组，发现在强化巩固治疗组中RUNX1-RUNXIT1^+^ AML的OS有比CBFβ-MYH11^+^ AML差的趋势（*P*＝0.062），诱导方案对患者OS和DFS的影响均不具有统计学意义。

195例CBF-AML患者中有93例患者合并KIT突变，合并KIT突变的患者CR率为95.7％。KIT突变位置主要包括exon8、exon17两种类型，CR率分别为100％和93.5％。KIT exon17突变主要涉及D816、N822两个突变热点，CR率分别为93.1％和95.7％。对190例达CR_1_状态的CBF-AML患者进行预后分析，KIT突变阳性和阴性患者的3年DFS率分别为54.6％（95％ *CI* 44.9～64.6）和75.2％（95％*CI* 67.9～84.9）。KIT突变是影响CBF-AML患者DFS的危险因素［*HR*＝1.929（95％*CI* 1.207～3.085），*P*＝0.006］（图1A），根据KIT突变类型进行亚组分析，仅exon17突变对CBF-AML患者DFS影响显著［*HR*＝2.124（95％ *CI* 1.336～3.375），*P*<0.001］（图1B）。KIT exon17突变中只有D816位点突变对DFS影响具有统计学意义（*P*＝0.022）（图1C）。尽管两种CBF-AML亚型患者的DFS差异无统计学意义，但根据亚组分析，KIT突变仅对RUNX1-RUNXIT1^+^ AML患者的DFS有影响。RUNX1-RUNXIT1^+^ AML患者中KIT突变阳性和阴性患者的3年DFS率分别为49.4％（95％ *CI* 37.7％～62.4％）和77.7％（95％ *CI* 69.7％～89.2％），合并KIT突变的患者DFS显著缩短［*HR*＝2.652（95％ *CI* 1.505～4.672），*P*＝0.001］（图2A）。此外，RUNX1-RUNXIT1^+^ AML患者中也只有KIT exon17突变是影响DFS的危险因素［*HR*＝2.880（95％ *CI* 2.21～6.60），*P*<0.001］（图2B），但除了KIT exon17 D816位点突变，N822位点突变也对DFS影响具有统计学意义（*P*值分别为0.005和0.022）（图2C）。相反，KIT突变不影响CBFβ-MYH11^+^ AML患者的DFS。KIT突变还与RUNX1-RUNXIT1^+^ AML较差的OS相关，但与CBFβ-MYH11^+^ AML的OS不相关，不同KIT突变类型对OS和DFS的影响相同。此外，我们将KIT突变比例以25％为界，分为KIT突变低比例组（low）和高比例组（high），未发现两组OS和DFS有明显差异（OS，*P*＝0.612；DFS，*P*＝0.377）。

**图1:**
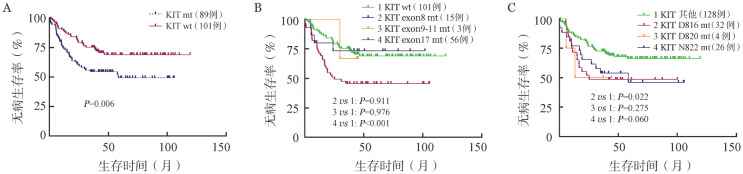
KIT基因突变状态及类型对核心结合因子相关急性髓系白血病患者无病生存的影响 A：不同KIT突变状态；B：不同KIT突变类型；C：不同KIT exon17突变位点。mt：突变型；wt：野生型

**图2:**
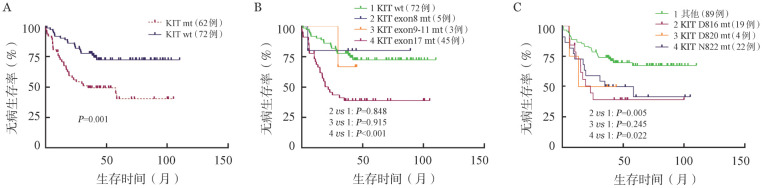
KIT基因突变状态及类型对RUNX1-RUNXIT1^+^ 急性髓系白血病患者无病生存的影响 A：不同KIT突变状态；B：不同KIT突变类型；C：不同KIT exon17突变位点

四、预后分析

按照CBF-AML总体和不同CBF-AML亚型分别对可能影响DFS的临床因素及基因突变进行单因素分析，将单因素分析中*P*值<0.1的变量纳入Cox模型中进行多因素分析。CBF-AML总体多因素分析结果显示，KIT exon17突变是影响患者DFS的独立危险因素［*HR*＝2.257（95％ *CI* 1.408～3.617），*P*＝0.001］，进行allo-HSCT是DFS的保护因素［*HR*＝0.502（95％ *CI* 0.293～0.859），*P*＝0.012］（表2）。RUNX1-RUNXIT1^+^AML多因素分析的结果与CBF-AML总体一致，因CBFβ-MYH11^+^ AML病例数较少，多因素分析未发现具有统计学意义的因素（表2）。

**表2:** 影响190例核心结合因子相关急性髓系白血病（CBF-AML）患者无病生存的多因素分析

因素	CBF-AML			RUNX1-RUNXIT1^+^AML			CBFβ-MYH11^+^AML		
	*HR*	95％ *CI*	*P*值	*HR*	95％ *CI*	*P*值	*HR*	95％ *CI*	*P*值
年龄≥45岁	1.512	0.929～1.460	0.096	1.504	0.847～2.673	0.164			
KITexon17突变	2.257	1.408～3.617	0.001	2.996	1.714～5.234	<0.001			
allo-HSCT	0.502	0.293～0.859	0.012	0.425	0.222～0.812	0.010			
+22异常							0.179	0.024～1.344	0.094
FLT3-TKD突变							2.058	0.784～5.401	0.143
WT1突变							2.533	0.839～7.652	0.099

注：allo-HSCT：异基因造血干细胞移植

五、allo-HSCT对KIT突变和染色质修饰相关突变患者的预后价值

鉴于KIT基因突变在CBF-AML患者中对预后的不良影响，我们分析了其在不同治疗组中的表达情况和预后意义，结果见图3A。在CBF-AML总人群中，按治疗方案分组，发现在行强化巩固治疗的患者中，KIT突变阳性的患者较KIT突变阴性患者DFS差（*P*<0.001）。而在行allo-HSCT治疗的患者中，未见KIT突变对患者DFS有不良影响。按是否合并KIT突变分组，发现在KIT突变阳性的患者中，接受allo-HSCT治疗的患者的DFS优于接受强化巩固治疗患者（*P*＝0.002）。但在KIT突变阴性的患者中，allo-HSCT治疗并不能改善患者的DFS。在RUNX1-RUNXIT1^+^ AML患者中，结果与此一致（图3B）。进一步证明了KIT突变是影响患者DFS的独立危险因素，接受allo-HSCT能明显改善这部分患者的预后。

**图3:**
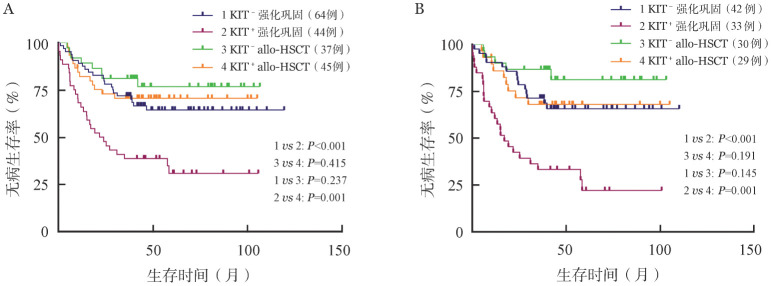
核心结合因子相关急性髓系白血病（CBF-AML）患者以KIT基因突变状态结合治疗方案分组各组的无病生存率比较 A：CBF-AML患者；B：RUNX1-RUNXIT1^+^ AML患者

由于本研究中染色质修饰突变和JAK突变仅在RUNX1-RUNXIT1^+^ AML中发现，我们单独分析了染色质修饰突变和JAK突变在不同治疗组中对RUNX1-RUNXIT1^+^ AML预后的影响，结果见图4A、4B。尽管染色质修饰基因突变对RUNX1-RUNXIT1^+^ AML患者的DFS无明显影响，但在染色质修饰突变阳性的患者中，行allo-HSCT治疗的患者DFS优于行强化巩固治疗患者（*P*＝0.001）。而在染色质修饰突变阴性的患者中，allo-HSCT治疗并不能改善患者的DFS。表明allo-HSCT能改善合并染色质修饰突变的患者的预后。通过亚组分析我们还发现在行强化巩固治疗的患者中，合并JAK突变的患者DFS时间较长（*P*＝0.031）。

**图4:**
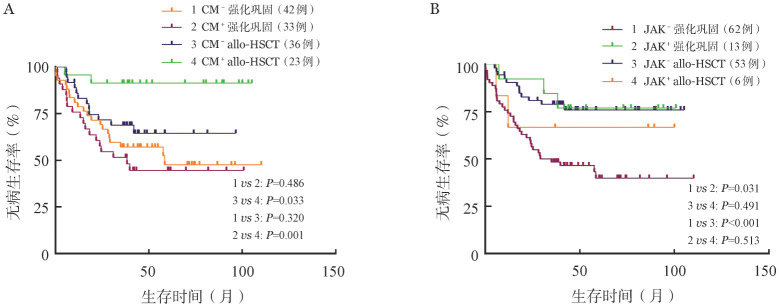
RUNX1-RUNXIT1^+^ 急性髓系白血病（AML）患者以染色质修饰（CM）和JAK基因突变状态结合治疗方案分组各组的无病生存率比较 A：染色质修饰；B：JAK

## 讨论

尽管CBF-AML具有相对较好的CR率和预后，但已有大量研究表明，不同CBF-AML亚型间存在异质性，CBFβ-MYH11^+^ AML患者的预后往往优于RUNX1-RUNXIT1^+^ AML患者[16]。我们中心的结果显示，在接受强化巩固治疗的患者中，RUNX1-RUNXIT1^+^ AML患者的OS有比CBFβ-MYH11^+^ AML差的趋势。最近一项研究显示，以中剂量阿糖胞苷为诱导治疗方案RUNX1-RUNXIT1^+^ AML的累积复发率、OS和RFS优于常规剂量阿糖胞苷方案[17]，我们的研究显示不同剂量去甲氧柔红霉素诱导治疗对患者的OS和DFS影响无明显统计学意义。

研究表明单一CBF-AML的特征性融合蛋白不足以导致白血病，需要其他驱动突变共同参与致病，已有小鼠模型证明受体酪氨酸激酶（如KIT和FLT3）中的协同突变可导致疾病发生[18]–[20]。然而，目前各种基因突变在CBF-AML中的预后意义尚不清楚。NCCN指南将具有KIT突变的患者归为中危组，并建议进行allo-HSCT治疗或临床试验。本研究表明，KIT、FLT3和RAS等驱动基因突变对RUNX1-RUNXIT1^+^ AML和CBFβ-MYH11^+^ AML患者的预后影响不同。

KIT突变在CBF-AML患者中的发生率最高，大部分研究认为其与不良预后有关。在RUNX1-RUNXIT1^+^ AML患者中，多数研究认为KIT exon17突变，尤其是KIT D816突变与复发率高、生存时间短相关，而在CBFβ-MYH11^+^ AML患者中，研究结果则显示KIT exon8突变与不良预后相关[12],[21]–[23]。我们的研究结果表明，KIT突变可显著影响RUNX1-RUNXIT1^+^ AML的OS和DFS，尤其是KIT exon17突变，且D816突变比N822更能代表其不良预后特征。相反，KIT突变不影响CBFβ-MYH11^+^ AML的预后，但考虑本研究中该组患者纳入数较少，应进一步扩大样本量检验。

FLT3突变主要有FLT3-ITD和FLT3-TKD两种类型，这两种类型突变累及约30％的AML患者[24]。FLT3-ITD突变在两种CBF-AML亚型的发生率均较低，约为5％；FLT3-TKD突变在CBFβ-MYH11^+^ AML比RUNX1-RUNXIT1^+^ AML高，分别为15％和5％[7],[12]。既往多数研究表明FLT3-ITD在CBF-AML中预后较差[25]–[26]。我们的研究显示其对CBF-AML患者预后无显著影响，与一项意大利的研究类似[16]。FLT3-TKD突变在CBF-AML中的预后意义不明，一些研究认为其预后好[25]，另一些研究则提示其预后差[12],[26]–[27]，我们的队列仅单因素分析显示FLT3-TKD与CBFβ-MYH11^+^ AML患者不良OS相关。因此，FLT3对CBF-AML患者的预后意义有待进一步研究。

RAS突变，包括NRAS和KRAS突变，在CBFβ-MYH11^+^ AML患者中的发生率显著高于RUNX1-RUNXIT1^+^ AML。RAS突变对CBF-AML的预后影响具有争议，大部分研究表明RAS突变不影响预后[28]，而部分研究显示伴有RAS突变预后较好或较差[8],[21],[29]。我们的研究未发现RAS突变对CBF-AML患者的预后意义。同时未发现其余克隆性突变与OS、DFS的相关性。

已有不少研究发现染色质修饰相关突变（如ASXL2、ASXL1）、JAK突变、黏蛋白复合物相关突变（如STAG2）仅在RUNX1-RUNXIT1^+^ AML中发生[11]。部分研究表明伴有染色质修饰和黏蛋白复合物相关突变的患者预后较差[8]。我们的研究发现虽然染色质修饰相关突变不影响预后，但是合并该突变的患者进行allo-HSCT有更长的生存期。JAK突变在RUNX1-RUNXIT1^+^ AML中发生率较低，很少有研究报道其预后意义。Illmer等[30]的研究发现JAK2突变在CBF-AML中的检出率为3.6％，合并JAK2V617F基因突变的患者有80％复发。在我们的研究中，通过对治疗方案进行亚组分析，发现在行强化巩固治疗组合并JAK突变的患者预后较好，这一结果可能与合并JAK突变的患者较少合并KIT突变有关。

本研究表明RUNX1-RUNXIT1^+^ AML和CBFβ-MYH11^+^ AML患者具有不同的分子遗传学特点。未来的研究可以集中在两种CBF-AML亚型共有途径以及特异性途径的分子基础上，开发出新的治疗方法以指导临床决策。我们的研究结果显示RUNX1-RUNXIT1^+^ AML患者伴KIT exon17突变预后差，而allo-HSCT能改善这部分患者的预后，伴有染色质修饰相关基因突变的患者进行allo-HSCT能达到最佳预后。提示allo-HSCT在治疗高危CBF-AML中具有重要价值。最后，针对这些途径进行药物评估以及将这些分子发现与临床试验相结合的转化研究可能会改善CBF-AML患者的治疗。

本研究为单中心、回顾性、非随机研究，由于缺乏初诊时RUNX1-RUNXIT1和CBFβ-MYH11融合基因转录水平、缺乏强化巩固化疗时的微小残留病数据、缺乏详细的免疫学资料、治疗方案的非统一性、随访时间限制以及转诊后失访，均会影响统计检验效能。有必要进行样本量更大的随机对照临床试验，验证上述因素的预后意义，以进一步优化CBF-AML的个体化治疗。
